# Nutritional Value and Antinutritional Factors of *Balanites aegyptiaca* Seed Oils and Cakes for Animal Feed: A Review

**DOI:** 10.1002/fsn3.70478

**Published:** 2025-06-19

**Authors:** Sawadogo Salam, Compaore Inoussa, Bazongo Patrice, Sere Firmin, Bere Sompagnimdi Fréderic, Hien Mipro

**Affiliations:** ^1^ Department of Environment and Forests/Institute for Environmental and Agricultural Research (DEF/INERA) Ouagadougou Burkina Faso; ^2^ Laboratory for Study and Research on Soil Fertility and Production Systems (LERF‐SP)/NAZI Boni University Bobo Dioulasso Burkina Faso; ^3^ Laboratory for Molecular Biology, Epidemiology and Monitoring of Food and Water Transmitted Bacteria and Viruses (LaBESTA) Joseph KI‐ZERBO University Ouagadougou Burkina Faso

**Keywords:** antinutritional factors, *Balanites aegyptiaca*, cakes, nutritional value

## Abstract

Recent research on animal nutrition focused on the use of plant‐based lipid and protein sources as alternatives to fish oil and fishmeal, with a particular emphasis on by‐products from oilseed processing. Several studies have been carried out on seed and derived products of *Balanites aegyptiaca* . Therefore, this paper aimed to review scientific knowledge on the nutritional value and antinutrients of *B. aegyptiaca* oils and cakes for animal feed. A literature search of articles using Google Scholar and Research4Life was conducted between May and November 2024, followed by the selection of relevant articles from the reference list of collected articles. Then, a content analysis was performed. Results showed that *B. aegyptiaca* seeds had high oil content (30%–60%) with both saturated and unsaturated fatty acids. Among these, oleic, linoleic, and palmitic acids were the main ones. The remaining cakes after oil extraction had high contents of protein (30%–40%), lipids (4%–9%), and minerals (5%–15%). Common antinutritional factors in cakes were phytates, saponins, tannins, and oxalates, the contents of which ranged from 37 to 194, 1.6 to 18, 0.0043 to 0.034, and 15 to 350 mg/100 g, respectively. This review found that these cakes have been used in feed for animals such as goats, sheep, and rats, but there is limited knowledge on that of aquatic animals. Concerning oil, it has been successfully used in feed for *Clarias gariepinus* and *Clarias anguillaris* . Considering its high nutrient content and low antinutrient levels, *B. aegyptiaca* cakes and oils could be used as feedstuffs in animal feed. However, future studies are required for their better valorisation in feed by investigating their effects on growth and nutrient digestibility for other important farmed animal species.

## Graphical abstract

*Balanites aegyptiaca* seeds are rich in oils ranging from 30% to 60%. This oil had both saturated and unsaturated fatty acids, of which oleic, linoleic, and palmitic acids were the main ones found. The remaining cakes after oil extraction had high contents of protein (30%–40%), lipids (4%–9%), and minerals (5%–15%). Common antinutritional factors in cakes were phytates, saponins, tannins, and oxalates. This allows its potential use in feed for farmed species of terrestrial and aquatic animals.

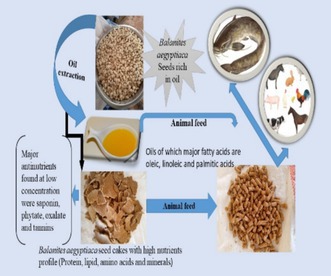

## 1 Introduction

*B. aegyptiaca* , known as the desert date, is a plant species from tropical and semi‐arid areas. It belongs to the family Zygophyllaceae (Balanitaceae). This species is of considerable socioeconomic importance and is distributed in the Sahelian, sudano‐sahelian zones, Arabian Peninsula, and India (Clement et al. 2011; Shackleton and De Vos 2022). It plays an important role in the pharmaceutical and cosmetic industries and for human and animal nutrition. Indeed, the most consumed parts of the plants are the fruits, being used for making natural juices (Amadou and Le 2017), the leaves as food for humans and animals, and the almonds for the production of edible oils, cosmetics, and vegetable milk (Alhassan et al. 2018). Other plant organs such as barks and roots are used in the pharmacopeia for the treatment of certain diseases (Chaudhry and Khoo 2004). Currently, the processing of oilseeds, especially those of *B. aegyptiaca* for oil production, is fast growing. This activity generates significant by‐products such as cakes, which can be cumbersome for the oil production industries. This has raised research questions from the scientific community regarding the sustainable use of *B. aegyptiaca* cakes in animal feed. Thus, several studies focused on the physicochemical parameters and biochemical compositions of almonds, oils, and cakes from the processing of seeds of local plant species, including *B. aegyptiaca* (Abdallah et al. 2012; Alhassan et al. 2018; Diedhiou et al. 2021; Amadou et al. 2023). These studies demonstrated that *B. aegyptiaca* almonds and derived cakes contained high levels of proteins and lipids. Currently, because of increased demand for feed ingredients and the high price of animal protein sources, there is a growing interest in finding other alternative protein sources, especially the plant‐based ones, which can be locally produced. In this context, cakes of *B. aegyptiaca* appeared to be one of these alternatives on the basis of its nutritional profile. However, cakes, like other protein sources of plant origin, contain indigestible fractions named antinutrients, the presence of which could limit their potential use in feed for animals by interfering with feed utilization (Annongu et al. 2009; Iyakutye et al. 2023). The major antinutrients found in *B. aegyptiaca* seed cakes were saponins, tannins, phytates, and oxalate (Samtiya et al. 2020). In addition, other studies focused on the incorporation of *B. aegyptiaca* cakes and oils in the feed for some terrestrial and aquatic animals in order to promote growth (Morkaz et al. 2011; Akourki et al. 2019; Sagne et al. 2019). However, few studies focused on the use of these oils and cakes with a particular emphasis on feed formulation on the basis of digestibility and nutrient bioavailability for aquatic animals and other terrestrial animals. Thus, there is a need to highlight our understanding and current knowledge of the nutrients and antinutrients composition of *B. aegyptiaca* oils and cakes in order to raise new research questions in feed formulation. Then, it appeared to be necessary to collect all available scientific information from the literature and to identify the various gaps for better consideration in future research regarding the use of these cakes and oils in animal feed. Therefore, this work aimed at reviewing scientific knowledge on the nutritional value, antinutritional factors, and current use of *B. aegyptiaca* oils and cakes in animal feed, including fish.

## 2 Review Methodology

A literature search was carried out by considering all published scientific works from 1980 to 2024 and related to biochemical composition of * B. aegyptiaca* oils and cakes and its use in animal feed by following recommendations of Rakita et al. (2023) with a slight modification on the type of research tools used. For this, articles were collected using Google scholar and Research4Life through AGORA with keywords such as press cakes, *B. aegyptiaca*, local vegetable oils, nutritional values, chemical composition, antinutritional factors, etc. The articles search was further extended to other databases such as PubMed and Web of science. Therefore, this allowed the collection of published articles which focused mainly on press cakes. Relevant papers were selected using exclusion criteria such as (i) use of artisanal oils, (ii) use of artisanal cakes, (iii) use of cake concentrate, and (iv) use of cake isolates. Indeed, the exclusion of articles was based on the type of oil extraction methods and derived cakes being employed in the study. This includes the quantity of produced oils and cakes, their cost, and availability for potential use as feedstuffs in feed for animals. The press cakes appeared to be cost‐effective and available protein sources which do not require other treatment before its inclusion into animal feed, compared to other cakes like artisanal cakes, concentrates, and isolates, which require more processing. Thus, articles using the press cakes in the study were considered to be relevant and then included for analysis in this review. Likewise, articles which used artisanal oils and cakes, cake concentrates, and isolates were considered irrelevant and then excluded. A search of other relevant papers was also performed by checking the references list from the collected articles. Finally, a content analysis of all collected articles was carried out by a careful reading of important sections including title, introduction, results, and conclusion. Then, data presented as results in articles were extracted and typed on Excel version 2016 and values of each considered variable were converted into the same unit according to the recommendation of Lykke and Padonou (2019). These data were then synthesized in tables by calculating the values range (minimum–maximum) for the contents of nutrients (protein, lipid, ash, carbohydrates, fiber, etc.) and antinutrient factors (phytate, tannins, oxalate, saponin, etc.).

## 3 Chemical Composition of *B. aegyptiaca* Oils

### 3.1 Physicochemical Characteristics of Seeds

The seeds of *B. aegyptiaca* were found to contain 30%–60% oil, and protein content ranged from 20% to 45% (Aviara et al. 2005; Tiétiambou et al. 2015; Alhassan et al. 2018). By considering almonds from other local plants, the protein content of *B. aegyptiaca* seeds could be more or less high compared to that observed for seeds of *Scleroclaria birrea* (29.5%), *Lannea kerstingii* (26.39%), and *Lannea microcarpa* (21.14%) (Bazongo et al. 2014; Ouilly et al. 2017; Sanou et al. 2024). The acid value refers to the content of the oil free fatty acids, which are products of hydrolysis of triglycerides. It is an important factor in assessing the quality of oils. Its low values indicate high stability of the oil. Thus, with regard to the oils of *B. aegyptiaca* , the acid value was found to vary between 0.4 and 1.56 mg KOH/g (Okia et al. 2013; MercyAkaagerger et al. 2016; Amadou et al. 2023; Bazongo et al. 2023). This showed that oils of *B. aegyptiaca* are more stable because of their low acid value compared to oils from other oilseed plant species, like *Sclerocaria birrea*, of which the seed oil's acid value was 6.56 mg KOH/g (Dadda'u et al. 2024).

### 3.2 Oils Fatty Acid Composition

The fatty acid content of *B. aegyptiaca* seed oil compared with other vegetable oils commonly used in feed is shown in Table 1. The most common fatty acids being found in the oil were linoleic acid, oleic acid, palmitic acid, and stearic acid, the contents of which ranged from 34% to 51%, 25%–38%, 13%–22%, and 10%–13%, respectively. In terms of saturation, *B. aegyptiaca* oil contained predominantly unsaturated fatty acids (72.55%–76.54%) compared to saturated ones (23.46%–27.45%). This confirmed findings of Souci et al. (2000) showing that vegetable oils have a low content of saturated fatty acids compared to that of mono and polyunsaturated fatty acids unlike oils of animal origin. Compared with other vegetable oils, *B. aegyptiaca* oils were found to contain more stearic, palmitic acids than those from soybean, sunflower and groundnut oils. This was similar considering the saturated fatty acids content of these seed oils. Thus, as fatty acids, especially saturated ones play an important role in nutrition, oil of *B. aegyptiaca* could be a good fatty acid source for animal feeding. Besides, oils of *B. aegyptiaca* contains some phytochemicals like phenolic compounds, antioxidants and some antinutrients at trace quantity which could be transferred from the seeds during the oil extraction process and providing health benefit as explained by Yadav et al. (2022). Regarding their low concentration, antinutrients might not have negative effects on nutrients absorption.

**TABLE 1:** Fatty acid composition of *B. aegyptiaca* oil compared with commonly used vegetable oils.

Fatty acids category	Fatty acid	Vegetable oils					Authors
		Balanites aegytiaca	Soybean	Palm	Sunflower	Groundnut	
Saturated	Stearic acid	10.29–13.34	3–3.53	3–6	3.35–5	2–3	Yousefi et al. (2012), Nile and Park (2013), Bazongo (2015), Ayoola et al. (2016), Aliyu et al. (2017), Karmakar et al. (2017), Diedhiou et al. (2021), Amadou et al. (2023), Bazongo et al. (2023), Bilska and Krzywdzinska‐Bartkowiak (2024)
Monounsaturated	Palmitic acid	12.8–22.02	10.62–12	37–41	6.34–7	10–11	
	Oleic acid	25.47–38.2	23.54–24	40–45	24.23–25	48–50	
Polyunsaturated	Linoleic acid	34.3–50.85	55–56.84	8–10	63.91–68	39–40	
SFA	Saturated Fatty Acid	23.46–27.45	15.25–16.89	32.45‐51.64	10.70–13.19	17.1–24.54	
UFA	Unsaturated Fatty Acid	72.55–76.54	83.11–84.75	48.36–55.35	86.81–89.31	57.2–57.42	

Generally, animals require fatty acids for their growth and other physiological needs. It is important to note that this requirement depends on the fatty acid category and the growth stage of the animal (Doppenberg and Van der Aar 2010). With regards to the oil of *B. aegyptiaca* , results showed high levels of UFA. In animal nutrition, it was reported that UFA had high digestibility and absorption rates than SFA (Rodriguez‐Sanchez et al. 2019). This confirmed that oil of *B. aegyptiaca* could be suitable as an energy or fatty acid source for animal feeding.

## 4 Chemical Composition of *B. aegyptiaca* Oilcakes

### 4.1 Proximate Composition

Cakes from processed *B. aegyptiaca* seeds have been widely studied in order to explore its use in animal feed. Thus, studies on the nutritional value of cakes were carried out as to determine its proximate composition. Results are summarized in Table 2. Findings from these studies demonstrated that these cakes contained high protein content (about 36%–54%). These results were in agreement with those of Mohamed et al. (2002), who also showed that cakes of *B. aegyptiaca* seeds had high protein levels. This was found to be higher than that of sunflower press cakes, the value of which ranged from 21.6% to 37.7% (Sobczak et al. 2020; Petraru et al. 2021). Similarly, high protein content of *B. aegyptiaca* cakes was observed compared to that from other conventional plant‐based protein sources such as soybean (44.03%), sesame (41.1%), cottonseed (24.4%), and peanut (43.5%) (Afaf and Sulieman 1999; Ahmed et al. 2009). These results showed that *B. aegyptiaca* seed cakes had protein contents comparable to those of several plant protein sources already used in animal feed. *B. aegyptiaca* seed cakes have fiber content ranging from 4.5% to 12.9%, which are lower than that of sunflower cakes (12.6% and 37%) (Kalmendal et al. 2011; Petraru et al. 2021). In this review, a wide range of nutrient content was observed, which could be attributed to the quality of seeds and the processing methods. This was further explained by García‐Rebollar et al. (2016), Petraru and Amariei (2020) who found that the chemical composition of oil seeds was related to the climatic conditions, agricultural practices, soil type, processing conditions, varieties, and growing conditions. Consequently, this variability may affect the quality and the amount of nutrients in the derived cakes, which are very important in feed formulation and animal feeding. This implies that feed formulators should be aware of this nutrient variability in order to provide supplemental inclusion of nutrients when the expected amounts in the cakes are not achieved. Considering the energy content of *B. aegyptiaca* seed cakes, it was found to be comparable to that of peanut cakes (2640 Kcal/kg), soybean cakes (2950 Kcal/kg) as reported by NIAS (2021) but lower than that of linseed cakes (4200 Kcal/kg) (Tarek‐Tilistyáka et al. 2014). For this reason, *B. aegyptiaca* cakes could be used for both high and low energy requirement animals such as poultry, cattle, and fish.

**TABLE 2:** Proximate composition of *B. aegyptiaca* cakes.

Nutrients	Range (%)	Authors
Dry matter	88.44–94.4	Mohamed et al. (2002), Ahmed et al. (2011), Ajayi and Ifedi (2014), and Akourki et al. (2019)
Crude protein	35.6–53.8	
Crude lipid	4.51–13.92	
Carbohydrate	8.28–32.59	
Fiber	4.01–12.89	
Total ash	5.04–15.38	
Energy (Kcal/kg)	1540.22–3174.8	

### 4.2 Amino Acid Composition

Most oilseeds and derived by‐products from seed processing contain amino acids that can improve feed quality. The amino acid composition of *B. aegyptiaca* seeds and cakes is presented in Table 3. Both seeds and cakes contained all amino acids. The presence of essential and non‐essential amino acids is noted with higher amino acid levels in cakes than those in seeds. This was explained by the fact that processing methods allowed for a better concentration of amino acids except that tryptophan can be denatured by the thermal effect (Muhammad et al. 2020). Indeed, the observed high or low concentration of amino acids could be linked to the hydrolysis of antinutritionnal components by the processing methods, allowing for release of more or less amino acids (Abdullahi et al. 2019). Further, seed pressing can be hot, which caused loss of certain amino acids by the heat treatment during the processing phase. According to NRC (2011), most vegetable ingredients have limited contents of lysine and methionine. However, these essential amino acids including others such as phenylalanine, valine, threonine, tryptophan, isoleucine, leucine, and histidine were identified in the seeds and cakes of *B. aegyptiaca* (Aremu et al. 2021). For instance, these cakes had higher amino acids content than those of common used ingredients in animal feed like soybean cakes of which contents of lysine, methionine, phenylalanine, valine, threonine, isoleucine, leucine, and histidine were 2.6 g/100 g, 0.43 g/100 g, 2.18 g/100 g, 2.01 g/100 g, 1.75 g/100 g, 2.22 g/100 g, 3.35 g/100 g, and 1.16 g/100 g, respectively (Singh et al. 2022). This shows that cakes of *B. aegyptiaca* can be considered as good amino acid sources for utilization in formulated plant‐based diets for animals.

**TABLE 3:** Amino acid composition (g/100 g protein) of *B. aegyptiaca* seeds and cakes.

Amino acids	Seeds	Cakes	Authors
*Essential amino acids*			
Leucine	5.00–7.30	7.62–8.60	Muhammad et al. (2018), Abdullahi et al. (2019), Aremu et al. (2021), Ogori, Eke, et al. (2022), and Ifedi et al. (2024)
Lysine	1.55–3.34	5.25–5.32	
Isoleucine	2.39–3.54	4.32–4.50	
Phenylalanine	2.97–4.08	3.99–5.15	
Tryptophan	0.84–1.37	1.02–1.18	
Valine	2.33–3.51	3.83–5.02	
Methionine	0.50–1.39	1.26–1.58	
Histidine	3.32–6.20	2.17–3.00	
Threonine	1.11–2.00	3.22–4.81	
*Non‐essential amino acids*			
Proline	3.45–3.55	3.86–4.01	
Arginine	1.38–6.19	5.25–8.07	
Tyrosine	3.44–3.61	3.10–4.09	
Cystine	1.45–3.39	0.91–1.52	
Alanine	2.28–3.49	3.83–5.02	
Glutamic acid	1.36–11.65	12.42–18.22	
Glycine	3.80–5.03	3.09–4.11	
Serine	3.94–4.00	3.67–4.82	
Aspartic acid	7.91–0.98	8.21–10.99	
Total amino acids	45.58–74.62	77.02–100.01	

### 4.3 Mineral Composition

The mineral composition of *B. aegyptiaca* cakes is presented in Table 4. The main minerals in these cakes were calcium, iron, potassium, and magnesium, with maximum levels being 6.05, 1.42, 14, and 6 mg/g, respectively. These contents were higher than those observed in peanut and soybean cakes, the calcium, iron, and magnesium contents of which were found to be 0.54 and 2.39 mg/g, 0.034 and 0.082 mg/g, and 1.97 and 2.59 mg/g, respectively (Longvah et al. 2017). These different minerals play an important role in human and animal nutrition through the regulation of intracellular electrolyte exchanges, muscle contractions, bone development, etc. (Jiang et al. 2002; Remington 2005). This highlighted that *B. aegyptiaca* cakes can be a good mineral source and used as feed ingredients. It is important to note that the bioavailability and absorption of minerals by animals vary, depending on the mineral nature, ingredients composition, and presence of antinutrients in feed ingredients. For instance, feeding animals with diets formulated with ingredients containing antinutritional factors like phytic acid may reduce some minerals uptake (Phosphate, Calcium) because it can be in a form of phytate‐mineral complexes that make these minerals unavailable for absorption by the animal (Chmielewska et al. 2020).

**TABLE 4:** Mineral composition of oil cakes.

Minerals	Range (mg/g)	Authors
Ca	0.49–6.05	Ajayi and Ifedi (2015), Yadav et al. (2022), and Ifedi (2023)
Cu	0.004–0.04	
Fe	0.004–1.42	
K	4.94–14.00	
Mg	1.70–6.00	
Mn	0.011–0.05	
Na	0.03–0.59	
Zn	0.03–0.43	

Furthermore, all presented data on the mineral content of *B. aegyptiaca* showed that its cakes meet the required amount for animals. Indeed, Cu, Zn, and Mn levels in these cakes were in the range of the required amounts of 0.005–0.008, 0.04, and 0.06 mg/g, respectively, for broiler chicken growth (NRC 1994). Levels of Ca, Mg, and iron were also in the typical requirement range of 0.002–0.0082, 0.0012–0.0018, and 0.03–0.050 mg/g for sheep (McDowell 2003). Considering aquatic animals, it was shown that most fish requirements for iron and Mg ranged from 0.03 to 0.17 and 0.4 to 0.6 mg/g, respectively (Lall and Kaushik 2021). Thus, feeding animals with *B. aegyptiaca* cakes would be beneficial in terms of meeting their dietary needs for growth and health.

## 5 Antinutritional Factors of *B. aegyptiaca* Seed Cakes

In order to ensure their survival, plants developed some defense mechanisms, especially production of secondary metabolites, to face predators such as insects and grazing animals (Iyakutye et al. 2023). These substances include phytate, saponin, oxalate, tannins, lectins, protein inhibitors, trypsin inhibitors, etc., which are stored in the plant organs (leaves, seeds and flowers); they are not involved in their growth process but act as protectors to animal and human actions. These metabolites are known as antinutritional factors (ANFs) that interfere with metabolism by affecting protein digestion, mineral utilization and then animal growth (Chothani and Vaghasiya 2011).

### 5.1 Composition of Antinutritional Factors

The contents of ANFs in *B. aegyptiaca* cakes are presented in Table 5. The concentrations of phytates, tannins, saponins, and oxalate in the cakes reached maximum levels of 194 mg/100 g, 0.034 mg/100 g, 18 mg/100 g, and 350 mg/100 g, respectively. These values varied among studies, due mostly to the type of processing techniques during oil production and the quality of the seeds used. Indeed, these factors lead to a variation in the composition of these elements, or even a significant reduction in the antinutritional factors content in cakes produced from the process (Lohlum et al. 2012). It was further proved that the contents of antinutritional factors appeared to be lower than those observed in the raw seeds, which were 7000 mg/100 g, 27.50 mg/100 g, and 427.90 mg/100 g for phytates, tannins, and saponins, respectively, except for levels of oxalate, which were 129 mg/100 g (Alhassan et al. 2018; Abdullahi et al. 2019). Moreover, it has been shown that *B. aegyptiaca* seed cakes contain lower phytic acid levels compared to soybean, sesame, and sunflower, and the content of this element ranged from 1000 to 5400 mg/100 g (Gupta et al. 2015).

**TABLE 5:** Composition of antinutritional factors in *B. aegyptiaca* cakes.

Antinutritional factors	Range (mg/100 g)	Authors
Phytate	37–194	Mohamed et al. (2002), Lohlum et al. (2012), and Ogori, Girgih, et al. (2022)
Tannins	0.0043–0.034	
Saponin	1.6–18	
Oxalate	15–350	

It is important to note that the use of any ingredients in feed for animals depends on their nutritional value. Thus, knowledge of the content and health or growth impacts of antinutritional factors in particular ingredients, especially plant‐based protein sources, is essential for better feed formulation.

### 5.2 Effects of Antinutritional Factors on Animals

Knowledge regarding the effects of antinutritional factors on animals allows us to pay particular attention to the use of certain plant ingredients in their diets. ANFs, by their nature, have effects on animals including terrestrial and aquatic ones. On the basis of their mode of action, these substances are classified into protein utilization inhibitors (saponins, tannins) and mineral utilization inhibitors (phytates, oxalate) (Samtiya et al. 2020). For instance, saponins were toxic to fish and can reduce feed consumption because of their bitter taste, whereas tannins were responsible for reducing protein solubility (Thakur et al. 2019). Phytic acids were found to interfere with nutrient absorption by making bonds with positively charged molecules under gastrointestinal pH conditions and consequently reduce nutrient digestibility for pigs and poultry (Woyengo and Nyachoti 2013). Similarly, oxalate forms complexes with minerals, especially calcium and magnesium, causing a their reduced utilization or bioavailability (Abara 2003; Ekpa and Sani 2018). Thus, these antinutritional factors have negative effects on animal growth and health when consumed in large quantities. The lethal doses of phytates, saponins, and tannins were 250–500 mg/100 g, 200 mg/kg, and 6% of animal weight, respectively (Bushway et al. 1998; Diwan et al. 2000; Asuquo and Etim 2011).

With regards to the *B. aegyptiaca* cakes, it is important to notice that values of all antinutritional factors appeared to be lower than the observed lethal doses. This leads to a conclusion that *B. aegyptiaca* cakes might not compromise both terrestrial and aquatic animals health when included in diets.

## 6 Use of *B. aegyptiaca* Oils and Cakes in Feed for Animals

### 6.1 Terrestrial Animals

Table 6 shows the growth performance of some animals fed with *B. aegyptiaca* seed cakes. A 13‐week study was conducted using two groups of lambs (initial live weight of 20 kg) fed with diets containing cotton seed cakes and *B. aegyptiaca* cakes. Animals fed diets made with *B. aegyptiaca* cakes at an inclusion rate of up to 20% indicated feed intake and weight gains of 1238 and 172 g/day, respectively. These results were similar between the two groups, meaning that *B. aegyptiaca* cakes could be used in feed as the cotton seed cakes. Likewise, experiments on Maradi red goats aged on average 7.5 months and fed a diet supplemented with 27% of *B. aegyptiaca* cakes also showed feed intake and weight gain of 384 and 18.9 g/day, respectively, which were higher than those observed in the control group fed commonly used diets. These results were similar to those observed by Elhadji Nouhou (2014) who used the seed cakes of *Piliostigma reticulatum* to feed Maradi red goats (initial weight of 9.16 kg) for 12 weeks.

**TABLE 6:** Feed consumption and growth of animals fed with *B. aegyptiaca* cakes.

Animals	Inclusion rate (Kg/100 kg of feed)	Growth performance (g/day)		Authors
		Food consumption	Weight gain	
Sheep	20	1238	172	El et al. (1983) and Akourki et al. (2019)
Goats	27	384	18.90	

The use of cakes from processed seeds of *B. aegyptiaca* in animal feed was studied and approved (Nour et al. 1985). Indeed, these cakes are highly rich in nutrients such as proteins, lipids, as well as trace elements such as minerals and amino acids, and their important role in the animal body has been demonstrated. For example, amino acids were found to be important for the proper functioning of the organism, as these elements are precursors for making proteins, nucleic acids, hormones, and neurotransmitters (Olaofe et al. 1994). In addition, the observed low fiber concentrations in *B. aegyptiaca* cakes allowed their potential use in feeds for monogastric animals, of which high fiber contents have negative effects on lipid metabolism, digestion, and growth (Kalmendal and Wall 2012).

Moreover, a study determined the effect of replacement of *B. aegyptiaca* cakes with groundnut cakes in feed on the cattle rumen environment and revealed that animals fed with diets at 5%, 10% and 15% replacement level had similar rumen gastric pH value, ammonia concentration, and number of bacteria (Morkaz et al. 2011).

Up to our knowledge, there are very few published data on the effect of incorporating *B. aegyptiaca* oils in diets on the performance of terrestrial animals. It is noted that the inclusion of this oil in diets at levels ranging from 10% to 20% in rat shows a similar growth rate and feed conversion rate between control group of rats (without oil) and those fed with oil‐based feed (Ifedi 2023). This showed that this inclusion has no negative effects on the growth of rats. In addition, it was shown that the remaining oils in cakes after oil production using seed are a source of energy in animal feed (Rakita et al. 2023). The same authors stated that fatty acids are vital for health and their presence in animal feed is necessary.

### 6.2 Aquatic Animals

Oils are energy sources and fatty acids are necessary for the growth of aquatic species. Fish oil has been the main oil used in fish feed because of its fatty acid content. However, there is a decline in its production because of overfishing, leading to an increased price of this resource. This led to the use of vegetable oils in fish feed. With regard to *B. aegyptiaca* oils and cakes, very few studies have been conducted with a focus on their inclusion in feed for aquatic species. The only studies, up to our knowledge, concerned fish from the Clarias genus. Thus, the effects of this oil on the growth of *C. anguillaris* larvae (initial weight of 0.33 g) were determined by Sagne et al. (2019). Fish were reared in tanks for 8 weeks, fed with diets containing 0%, 1%, 2%, 3%, and 4% of *B. aegyptiaca* oil. Results showed that an incorporation of 2% of this oil in the diet allowed for a better growth rate and feed conversion of 1.22%/day and 0.33, respectively. Furthermore, Sourabié et al. (2019) compared the growth performance of *C. gariepinus* (initial mean weight of 16.2 g) fed diets supplemented with different vegetable oils for 6 weeks. It was concluded that fish fed a diet with *B. aegyptiaca* oil exhibited a better growth rate (4.39%/day) compared to those fed diets supplemented with cottonseed oils, palm oils, and shea butter. This implies that *B. aegyptiaca* oils could be supplemented in catfish diets, although more detailed data are needed to conclude its inclusion in feed for other aquatic species used in aquaculture. On the basis of the oil quality for consumption, Bazongo (2015) compared the nutritional value of local seeds from 12 plant species and found that *B. aegyptiaca* belongs to the best plant species of which oils contain high levels of polyunsaturated fatty acids (oleic and linoleic acids) and essential fatty acids, necessary for animal feed formulation. Indeed, the UFA content of *B. aegyptiaca* oil (72.9%) was higher than that of other important oils such as coconut oil (0.38%), sesame oil (21.08%), and sunflower seed oil (23.53%) (Bazongo 2015; Kotecka‐Majchrzak et al. 2020). Thus, oils of *B. aegyptiaca* are a good fatty acid source for both animal and human nutrition.

Regarding cakes of *B. aegyptiaca* , its use in feed for farmed aquatic animals remains poorly documented and limited to a few species. Up to our knowledge, trials were conducted on *C. gariepinus* (initial weight of 27 g) by Ifedi (2023) using diets containing different inclusion levels of *B. aegyptiaca* cakes. It was shown that an incorporation rate of 30% of *B. aegyptiaca* cakes allowed for fish better growth and feed conversion rates of 1.36% and 1.2%, respectively. This indicates the potential use of these cakes in aquaculture feeds, although similar studies are needed for testing their effects on other aquatic species used in aquaculture.

## 7 Conclusion

*B. aegyptiaca* cakes are valuable sources of protein and micronutrients in terms of their chemical composition and currently approved by scientific data as feed ingredients for a limited number of animals such as goats, sheep, rats, and also fish like *C. gariepinus* . Considering the oils, they are an important source of unsaturated fatty acids. Regarding antinutritional factors, it is noted that the main ones were phytates, saponins, oxalates, and tannins. However, their contents appeared to be low, which makes the cake suitable for animal nutrition because less content of ANFs is associated with more available nutrients for absorption. Further, the observed low levels of fibers in these cakes, combined with their good nutrient profile, also appeared to be an advantage for its use in animal feed. All these data suggest a widespread valorization of *B. aegyptiaca* cakes and oils in the diet for other farmed species of terrestrial and aquatic animals. This could help the feed industry to extend the supply of plant protein sources, protein substitutes, and to reduce the discharge of wastes into the environment. However, it was revealed that there is nutrient variability in the cakes, which could be a constraint for animal nutritionists to get a standardized ingredient during feed formulation. Thus, it is recommended to oil‐producing industries to improve their extraction methods to provide products with less variable nutrient content for the animal feed industry. Furthermore, the available data regarding the use of these oils and cakes in diets for animals and their effects on growth and nutrient utilization appeared to be insufficient in terms of considering some nutritional parameters such as nutrient digestibility and bioavailability over various types of animals. Therefore, in order to provide in‐depth knowledge on the supplementation of *B. aegyptiaca* oils and cakes in diets for animals, further potential future studies are required. These should emphasize the valorization of these oils and cakes in feed by performing standardized studies on nutrient utilization and digestibility and a broad species testing from both the livestock and aquaculture sectors.

## Author Contributions

**Sawadogo Salam:** conceptualization, writing – original draft, visualization, writing – review and editing. **Compaore Inoussa:** conceptualization, supervision, validation, writing – review and editing. **Bazongo Patrice:** conceptualization, writing – review and editing. **Sere Firmin:** conceptualization, writing – review and editing. **Bere Sompagnimdi Fréderic:** conceptualization, writing – review and editing. **Hien Mipro:** conceptualization, writing – review and editing, supervision.

## Conflicts of Interest

The authors declare no conflicts of interest.

## Data Availability

The data that support the findings of this study are available on request from the corresponding author. The data are not publicly available due to privacy or ethical restrictions.
